# Can Forgetting Be Due to Changes in Engram Cell Plasticity?

**DOI:** 10.3389/fnbeh.2022.945985

**Published:** 2022-07-07

**Authors:** Pascale Gisquet-Verrier

**Affiliations:** NeuroPSI—Institut des Neurosciences Paris-Saclay, Centre CEA Paris-Saclay, Saclay, France

**Keywords:** forgetting, engram cells, consolidation hypothesis, synaptic strength, memory reactivation

## Introduction

In a recent paper, Ryan and Frankland (2022) presented a very interesting paper on forgetting, trying to combine classical ideas and a number of considerations rarely discussed in this area (but see Miller, 2021). Obviously, forgetting processes should be highly related to encoding memory processes. Adopting the consolidation hypothesis, a widely accepted leading hypothesis, Ryan and Frankland proposed that learning induces increases in the synaptic strength between cells active during encoding. They further considered that memories are stored in these ensembles of neurons that are also termed engram cells. Based on that scheme, they proposed that forgetting results from a decrease in the synaptic strength, reducing engram cell accessibility during retrieval. They further suggested that a form of adaptive engram cell plasticity could account for the reversibility of forgetting.

Their new view mainly relies on a series of studies in which engram cells were manipulated using immediate early gene-based tagging with recent and sophisticated methods (Liu et al., 2012; Josselyn et al., 2015; Ryan et al., 2015; Josselyn and Tonegawa, 2020).

To summarize briefly, in these studies the authors used a contextual fear conditioning paradigm to establish a memory. In a typical experiment, mice placed in a particular context received a series of electrical footshocks during which active neuronal cells within the CA1 area of the hippocampus (engram cells) were tagged using optogenetic and chemogenetic methods. During retrieval, mice were replaced in the training context and retrieval was evaluated through their freezing responses, indicating that fear to the training context had been encoded. From these studies, it was postulated that (1) training induces protein dependent synaptic changes between the engram cells, resulting in potentiated synaptic connections between them, (2) direct activation of these engram cells can induce the expression of memory (fear), and (3) preventing normal functioning of these engram cells during encoding prevents the retrieval of memory.

In another study, Ryan et al. (2015) further showed that a post-training protein synthesis inhibitor delivered in CA1 prevents synaptic changes in engram cells, and induces retrograde amnesia: mice were no longer able to express fear when replaced in the training context. However, the authors showed that the artificial activation of the engram cells (tagged during training) was able to reinstate the retrieval of memory, suggesting that protein synthesis is necessary for retrieval and not for encoding processes.

However, a thorough analysis of these data leads to three main conclusions, leading to alternative views questioning the consolidation hypothesis, and more importantly for our purpose, calls into question Ryan and Frankland's view on forgetting.

## New Memories Do Not Require Any New Protein Synthesis

As previously noted, Ryan et al. (2015) demonstrated that protein synthesis is not necessary in order to establish the memory, a result confirmed by other studies (see Gisquet-Verrier et al., 2015; Gisquet-Verrier and Riccio, 2018). Such a conclusion is at odds with the traditional and dominant consolidation hypothesis claiming that new protein synthesis is a necessary condition to induce connection increases within the synaptic network presumed to support the memory. This result reported by Ryan et al. (2015) is more in agreement with the view that amnesic treatments disrupt memory through a form of state-dependent processes. Accordingly, amnesic treatments delivered at the time of training, even when administered within the brain, do not prevent the formation of memory but modify the internal state of the animal. As a consequence, due to a form of state dependency, the retrieval is disrupted at the time of testing, when the subject has returned to a normal state. The best evidence supporting this hypothesis is provided by repeated studies demonstrating that amnesic treatments delivered again shortly before testing reinstate retrieval of the initial information (see Gisquet-Verrier and Riccio, 2018, 2019).

This result strongly argues against a major aspect of the classical conceptual framework of consolidation which is considered as a time- and protein-dependent process aimed at transforming an episode into a stable memory. The findings further indicate that protein-dependent changes induced by training within the engram cell network are not necessary to establish and retrieve a memory.

## Potentiated Synaptic Connections Should Not be Considered as the Support of Memory

Considerable evidence shows that training induces protein-dependent changes in synaptic connections within the activated neuronal network. Such a result has been analyzed as the main evidence indicating that the involved neuronal network constitutes the support of memory.

However, increases in synaptic connections can be obtained in various circumstances in which a particular neuronal network was significantly activated, such as after epileptic crisis or electrical brain stimulations (see Gisquet-Verrier and Riccio, 2019), indicating that they do not appear to be specific to memory processes. In a recent paper, Abraham et al. (2019) provide evidence indicating that plasticity of synapses might not be considered as the mechanism of long-term memory. As previously noted, it has been experimentally established that memories can be correctly encoded, even in the absence of any synaptic changes (Ryan et al., 2015). A parallel between consequences of training on synapses and exercise on muscles can be drawn. Exercise makes the involved muscles grow, and the direct stimulation of these muscular fibers may possibly trigger the reproduction of the action, but the action in itself is not registered in the fiber of the muscle. Hence, changes in connections resulting from training experience might be independent of its representation and cannot be firmly considered as the support of memory. Accordingly, the tagging of engram cells is not required for memory retrieval and thus should not be involved in forgetting mechanisms.

## Training Induced Potentiated Synaptic Connections are Not Necessary for Retrieval

Supporting our alternative view, it has been shown that when potentiated synaptic connections between the engram cells was prevented by a protein synthesis inhibitor, it was possible to obtain a correct recall of memory through a direct stimulation of the engram cells (Ryan et al., 2015). Curiously, the authors proposed that the protein synthesis and thus the subsequent changes in synaptic connections, were not necessary to establish the memory but were required for retrieval. However, even according to their view, it is difficult to understand how a direct stimulation of engram cells can reactivate a neural network while, due to the absence of protein synthesis, the tagging of the engram cells was not established during training. In addition, such a view is also unable to explain why delivering again a protein synthesis inhibitor just before testing can reinstate the memory (Gisquet-Verrier et al., 2015).

This latter case demonstrates that the memory can be accessible at the time of retrieval without any new protein synthesis nor synaptic changes between training and testing. Numerous evidence provided by many studies in the 70's (see Gisquet-Verrier and Riccio, 2018, 2019; Miller, 2021) indicated that recovery of memory after amnesic treatments may also be achieved by a pretest exposure to a reminder (reinforcer, conditional or discriminative stimuli, experimental context). Importantly, reminders have also been repeatedly shown to reverse spontaneous forgetting (see Gisquet-Verrier and Riccio, 2012). In all, these studies indicate that forgetting, as in the example of post training amnesia, results from difficulties in retrieving previous information. Forgetting may be alleviated in different ways: through a reactivation induced by a reminder (referring to external or internal contextual cues) or by the direct stimulation of their internal representations, i.e., cells activated during training.

Hence, reactivating a part of the memory (either by a direct reactivation of the “engram cells” or through the use of a reminder) is able to trigger the reactivation of the whole memory, even in the absence of any changes within the neuronal network engaged during training. Once again, these results strongly suggest that forgetting is due to retrieval difficulties that are not related to engram cell plasticity.

## Conclusion

In their perspective paper, Ryan and Frankland (2022) provide a thorough and comprehensive analysis of forgetting through an extensive review of the literature. They provide extensive evidence differing from the traditional consolidation view, especially concerning the reversibility of amnesia and forgetting. For these reasons, their paper makes undoubtedly an important contribution in the field However, they also provided a view in substantial agreement with the consolidation model, which has dominated the literature for more than 60 years and become a dogma.

In agreement with this view, the authors proposed that memory storage requires a protein-dependent process, during which the synaptic strength within the neuronal network that was engaged during training (engram cells), increases. However, we provided arguments indicating that (1) storage and retrieval of memory can be obtained in the absence of any new protein synthesis necessary to tag the engram cells and (2) changes in synaptic connections, a protein synthesis dependent process, are not specific to a training/memory situation. These two considerations not only question the consolidation view, as previously underlined (Gisquet-Verrier and Riccio, 2016, 2018, 2019) but also the forgetting hypothesis recently proposed by Ryan and Frankland (2022). Is it possible to consider that forgetting results from changes in synaptic plasticity of the neuronal network engaged during training when evidence suggests that engram cells are not the support of memory?

Forgetting is not a definitive process, and, as underlined by Ryan and Frankland, forgetting can be reversible. Interestingly, a forgotten memory can be retrieved with the help of a reminder and be restored by reproducing a state close to the one prevailing during training or shortly thereafter (Winocur et al., 2009; Miller, 2021; Gisquet-Verrier and Riccio, 2022). We all know that information unavailable at a particular time may come to mind some time later. Considerable evidence indicates that the retrievability of a particular memory is strongly influenced by the surrounding context. Forgetting corresponds to a transient lack of retrievability which might be definitive when the subject does not find any cue able to reactivate the target memory. Accordingly, forgetting might be viewed as a highly flexible and dynamic process and it seems quite unlikely that such a variable and rapid process could be sustained by a form of engram cell plasticity requiring long and complex adaptive processes.

We showed that such a view is not in agreement with many results in the literature (even those provided by the authors themselves) and is not in agreement with what memory is: a dynamic and fast process (Hebscher et al., 2019), highly flexible, with partial, transient and reversible forgetting. It thus seems timely to take into account all these elements, to revisit the consolidation concept, and to investigate other forms of possible memory representations (see Abraham et al., 2019) that should be involved in forgetting processes.

## Author Contributions

The author confirms being the sole contributor of this work and has approved it for publication.

## Conflict of Interest

The author declares that the research was conducted in the absence of any commercial or financial relationships that could be construed as a potential conflict of interest.

## Publisher's Note

All claims expressed in this article are solely those of the authors and do not necessarily represent those of their affiliated organizations, or those of the publisher, the editors and the reviewers. Any product that may be evaluated in this article, or claim that may be made by its manufacturer, is not guaranteed or endorsed by the publisher.
